# Glycosylation site occupancy in health, congenital disorder of glycosylation and fatty liver disease

**DOI:** 10.1038/srep33927

**Published:** 2016-10-11

**Authors:** Andreas J. Hülsmeier, Micha Tobler, Patricie Burda, Thierry Hennet

**Affiliations:** 1 Institute of Physiology, University of Zürich, Winterthurerstrasse 190, 8057 Zürich, Switzerland; 2 Division of Metabolism and Molecular Pediatrics, University Children’s Hospital Zürich, Steinwiesstrasse 75, 8032 Zürich, Switzerland

## Abstract

Glycosylation is an integral part in health and disease, as emphasized by the growing number of identified glycosylation defects. In humans, proteins are modified with a diverse range of glycoforms synthesized in complex biosynthetic pathways. Glycosylation disorders have been described in congenital disorders of glycosylation (CDG) as well as in acquired disease conditions such and non-alcoholic fatty liver disease (NAFLD). A hallmark in a subset of CDG cases is the reduced glycosylation site occupancy of asparagine-linked glycans. Using an optimized method protocol, we determined the glycosylation site occupancy from four proteins of hepatic and lymphatic origin from CDG and NAFLD patients. We found variable degrees of site occupancy, depending on the tissue of origin and the disease condition. In CDG glycosylation sites of IgG2 and IgA1 were occupied to normal levels. In NAFLD haptoglobin and transferrin glycosylation sites were hyper-glycosylated, a property qualifying for its use as a potential biomarker. Furthermore, we observed, that glycosylation sites of liver-originating transferrin and haptoglobin are differentially occupied under physiological conditions, a further instance not noticed in serum proteins to date. Our findings suggest the use of serum protein hyperglycosylation as a biomarker for early stages of NAFLD.

Alcoholic liver disease (ALD), non-alcoholic fatty liver disease (NAFLD) and congenital disorders of glycosylation (CDG) share common symptoms manifested by the development of fatty liver, liver fibrosis/cirrhosis and insulin resistance^1^. Whereas CDG constitutes a group of autosomal recessive inherited diseases, ALD and NAFLD are considered as acquired disease conditions^2^,^3^. Although, a recent study of twins based on MRI assessments suggests that hepatic steatosis and fibrosis are heritable traits^4^. NAFLD can be grouped into benign liver steatosis and the more progressed and inflammatory form of non-alcoholic steatohepatitis (NASH). NAFLD/NASH is also been described as the manifestation of the metabolic syndrome in the liver^1^. A recent report describes NASH as a preceding determinant for the development of the metabolic syndrome with potential implications on the clinical diagnosis and treatment^5^. The search of biomarkers for non-invasive diagnosis, addressing the prevalence and the scope of clinical presentations is a major focus in NAFLD research^6^. ALD and NASH share common traits, such as the occurrence of Mallory-Denk bodies in the cytoplasm of liver cells, upregulation of the cytochrome P2E1 with subsequent increase in reactive oxygen species and accumulation of 4-hydroxy-2-nonenal in the liver tissue. The accumulation of 4-hydroxy-2-nonenal is made responsible for the development of hepatocellular carcinoma in late stage disease conditions. For the differentiation of ALD and NASH non-invasive diagnostic measures are lacking and liver biopsies are required for diagnosis^7^. Serum values of aminotransferases and gamma-glutamyl transpeptidase and the mean corpuscular volume of erythrocytes are overlapping between NASH and ALD samples. Nevertheless, a direct comparison of levels of carbohydrate deficient transferrin (CDT) in serum can be used to differentiate between NASH and alcoholic hepatitis patients^8^. N-linked glycosylation profiles have been used for diagnosing liver cirrhosis and to differentiate patients with hepatocellular carcinoma from cirrhotic patients^9^,^10^. Accordingly, an increase of a-galactosylated N-glycans with concomitant decrease of the galactosylated glycoforms serum samples, and in the Fc-region of serum IgG has been proposed as a biomarker for diagnosing advanced NASH related fibrosis and differentiating between liver steatosis and NASH^11^,^12^.

CDG is a multi-systemic condition affecting various glycosylation pathways. A new nomenclature addressing CDG forms deriving from differing glycan biosynthetic pathways was proposed, using the official gene symbol of the protein involved followed by “-CDG”^13^. A subset of CDG forms derived from the N-glycan biosynthesis typically display reduced glycosylation site occupancy of secreted proteins. The reduced glycosylation frequency is due to gene defects of enzymes mediating the assembly of the precursor dolichol-linked oligosaccharide or the oligosaccharide transfer to the newly synthesized glycoprotein. Other forms of CDG display aberrant glycan structures, but normal glycosylation frequency on secreted proteins, due to gene defects in proteins involved in the glycan maturation and processing in the Golgi. A common symptom to CDG and ALD is a reduced N-glycosylation site occupancy, and is characterized by an increase of CDT in the blood of affected patients^14^. CDT levels are routinely assessed by isoelectric focusing gel electrophoresis, HPLC analysis or liquid chromatography coupled mass spectrometry (LC-MS)^15^,^16^,^17^. We have previously developed a multiple reaction monitoring mass spectrometric (MRM-MS) assay to directly determine the N-glycosylation site occupancy at the peptide level, and found decreasing site occupancy in correlation with the severity of the clinical symptoms in the respective CDG forms^18^. Here we devised an optimized protocol, omitting immunoaffinity purification and simplified sample preparation procedures for the semi-quantitative determination of the N-glycosylation site occupancy of four serum proteins of hepatic and lymphatic tissue origin. We validated this protocol using a serum cohort from CDG patients with known hypoglycosylation phenotypes and explored its use for determining potential biomarkers for NAFLD. We selected the serum glycoproteins transferrin, haptoglobin, IgG2 and IgA1 to compare the glycosylation status of proteins derived from hepatic and lymphatic origin.

Serum IgG glycosylation has been explored in search for biomarkers for autoimmune and congenital disease conditions. Changes in the N-glycan structures at the glycosylation site in the conserved Fc-region of the IgG heavy chain have been shown to modulate the binding of IgG to Fc-gamma receptors and C-type lectins, thereby influencing the inflammatory responses in autoimmune conditions (reviewed in refs 19 and 20). In congenital defects, abnormal IgG N-glycosylation for classical galactosaemia and MAN1B1-CDG-specific IgG glycans were described recently^21^,^22^. The variable Fab-region of IgG and IgA can also contain glycosylated sequons and might have emerged via somatic hypermutation^19^,^23^. The significance of Fab-glycosylation of IgG in (patho-)physiology and immunity has been discussed recently^24^. IgA1 contains two N-glycosylation sites in the constant Fc region and up to six occupied O-glycosylation sites in the hinge region. GlcNAc terminating IgA1 glycans selectively bind to the transferrin receptor, mediating effector functions in IgA nephropathy. Whereas glycans terminating with galactose serve as ligands for the asialoglycoprotein receptor, mediating the clearance of IgA from the serum^23^. Aberrant glycosylation of IgA1 has been ascribed to result in the depositions in the glomeruli of the kidney mesangium in IgA nephropathy^25^.

Transferrin and haptoglobin are serum glycoproteins produced in hepatocytes. Haptoglobin contains four N-glycosylation sites and belongs to the acute phase proteins, whose blood levels and glycosylation profiles change during inflammatory reactions in sepsis, tissue damage or cancer^26^. Haptoglobin binds free hemoglobin for degradation in the liver and for iron recycling. The binding characteristic of haptoglobin to surface receptors of macrophages in the tumor microenvironment might play a crucial role for tumor cell growth and survival^27^. The iron-binding blood protein transferrin contains two N-linked glycosylation sites. Transferrin has been utilized since decades as a biomarker for monitoring alcohol abuse^28^. Carbohydrate deficient transferrin is characterized by a reduced N-glycosylation site occupancy or alterations in N-glycosylation profiles and is used as a versatile diagnostic marker in CDG research. Serum transferrin glycoforms are monitored by isoelectric focusing gel electrophoresis, HPLC or mass spectrometric approaches^16^,^18^,^28^,^29^.

## Results

Reduced site occupancy of N-linked glycosylation sites is a hallmark in CDG and ALD. Previously, we developed a MRM-MS based method for directly determining the site occupancy at the peptide level and found decreasing occupancy in correlation with the severity of the clinical symptoms displayed in CDG with reduced N-glycosylation frequency^18^. Here we devised a sample preparation protocol in favor of simplicity, omitting previous glycoprotein enrichment by affinity purification. One μl of serum was reduced and alkylated before trypsin digestion, and a single sample transfer was required for isolating the tryptic peptides using a cation exchange resin. N-linked glycopeptides were enzymatically de-glycosylated with PNGase F in ^18^O-water containing buffer, introducing the ^18^O-isotope to the newly formed aspartic acid residue after deglycosylation. Isotopic labeling of the glycosylation sites helps to differentiate PNGase F reaction products from spontaneous deamination reactions of unoccupied asparagine residues. C-terminally isotope labeled standard peptides corresponding to unglycosylated and deglycosylated glycopeptide sequences were added to the samples to assess the relative N-glycosylation site occupancy. One glycosylation site per glycoprotein was assayed, based on robust detection in LC-MS-MS peptide sequencing, using the Mascot peptide score algorithm (Table 1).

### Testing the linearity in the peptide detection

**Figure 1:**
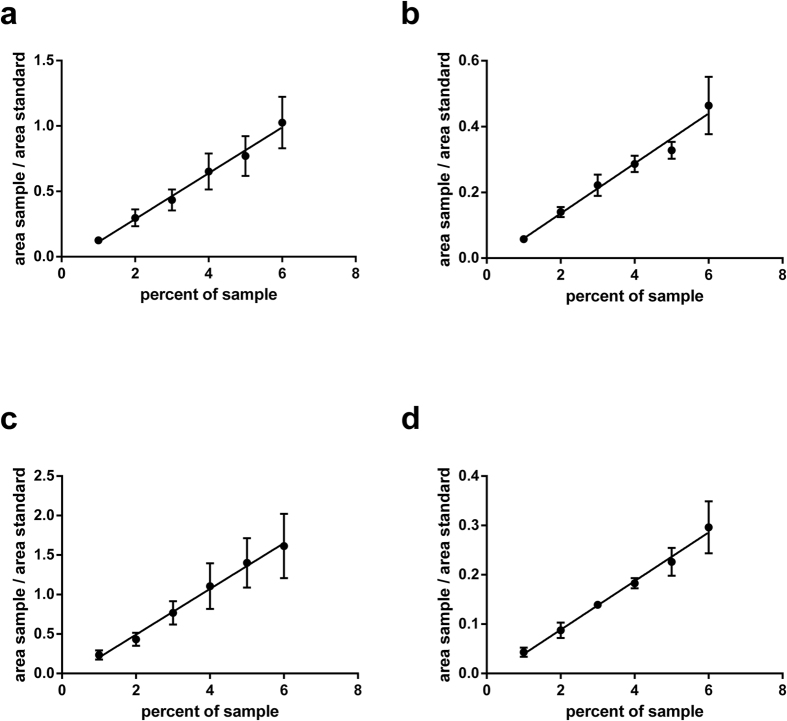
Linear relationship in the ratio of standard peptides with increasing amounts of sample. Increasing amounts of the sample preparation were dissolved with the standard peptide mix and subjected to LC-MRM-MS. The areas of the sample per area of the standard peptides are plotted against the amounts of sample. Data points are displayed as mean values from triplicate sample preparations, and the error bars indicate the standard error of mean (SEM) values. Linear regression analyses was performed for the deglycosylated transferrin (**a**), r^2^ = 0.99), haptoglobin (**b**), r^2^ = 0.98), IgA1 (**c)**, r^2^ = 0.99) and IgG2 peptides (**d)**, r^2^ = 0.99).

**Figure 2:**
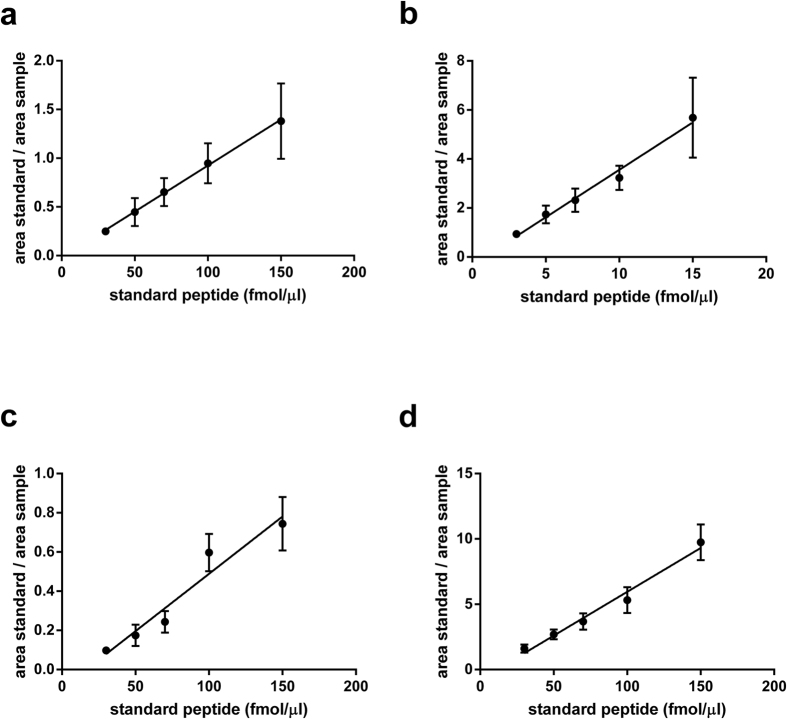
Linear relationship of the ratio of sample derived peptides with increasing amounts of standard peptides. Increasing concentrations of the standard peptide mix were added to the sample before subjecting to LC-MRM-MS (>70% purity, Supplemental Fig. 3). The areas of the sample per area of the standard peptides are plotted against the standard peptide concentrations. Data points are displayed as mean values from triplicate sample preparations, and the error bars indicate the standard error of mean values. Linear regression analyses was performed for the deglycosylated transferrin ((**a)**, r^2^ = 1.00), haptoglobin ((**b**), r^2^ = 0.99), IgA1 ((**c**), r^2^ = 0.94) and IgG2 peptides ((**d**), r^2^ = 0.98).

The linearity in the peptide detection was tested by loading increasing amounts of sample onto the nanobore column. We obtained a linear relationship of the amount of sample relative to the standard peptides when loading 1 to 6 percent of the sample preparation with r^2^ values of 0.98 and 0.99 after regression analyses (Fig. 1). Accordingly, we assayed for linearity with increasing amounts of standard peptides added to four percent of sample. We obtained a linear relationship with the standard peptides at concentrations from 25 to 150 fmol/μl for the transferrin, IgA1 and IgG2 peptides. The corresponding r^2^ values were calculated between 0.94 and 1.00 after regression analyses. For the haptoglobin peptides a 10-fold lower concentration was used in the standard peptide mixture, due to higher signal intensities observed for this peptide (Fig. 2). For the linearity tests technical triplicates were necessary to obtain a precise linear fit. Therefore, three replicates starting with the trypsin digests were prepared for each serum sample analyzed.

### Occurrence of spontaneous deamination

The occurrence of spontaneous deamination of asparagine was detected by the presence of ^16^O-aspartic acids in the glycosylation sequon. We observed a varying degree of deamination reactions, depending on the peptide sequence ranging from 2.9% in the haptoglobin peptide to 12.9% in IgA1 throughout all samples analyzed (Supplemental Table 2). Since the deglycosylation reaction was performed in ^18^O-containing buffer, ^16^O-aspartic acid within the sequon must have formed prior PNGase F mediated deglycosylation. Therefore, these values add towards the unglycosylated peptide count. For the IgG peptide only ^18^O-asparic acid could be detected, indicating that the amount of ^16^O-water in the reaction buffer is low and evades detection with the instrumentation used in this study (Table 2). Therefore, it appears likely that the content of ^16^O-water in the buffer used for the deglycosylation reaction did not contribute significantly to the ^16^O-aspartic acid counts.

### Serum samples

**Figure 3:**
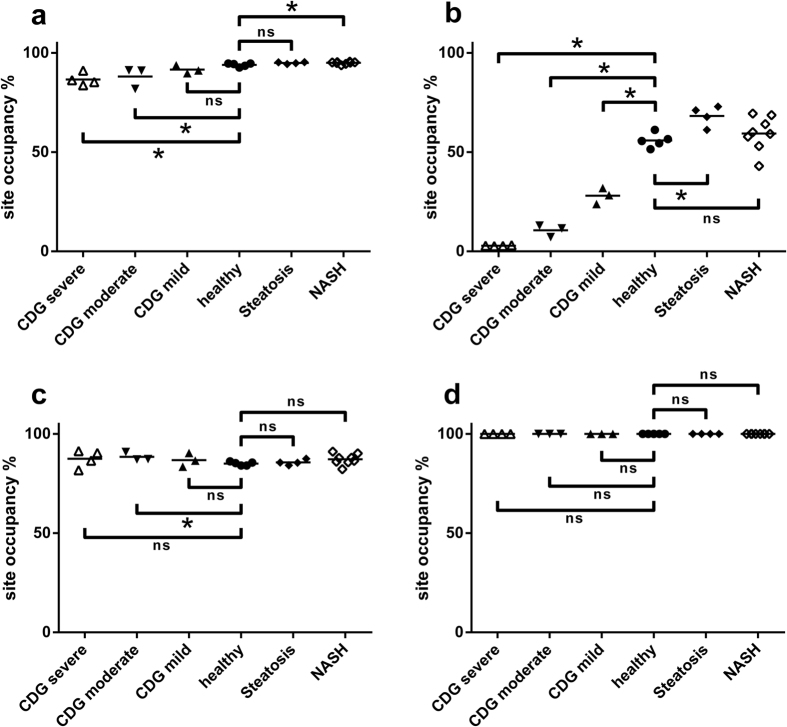
N-glycosylation site occupancy in CDG and NAFLD. The site occupancy in % of the transferrin (**a**), haptoglobin (**b**), IgA1 (**c**) and IgG2 (**d**) glycopeptides is plotted for the CDG, NAFLD and healthy control samples. Each data point represents the mean value of triplicate measurements starting with the sample preparation. The biological mean values with error bars (SEM) are indicated in each group of serum samples plotted. Differences between groups were calculated using the Mann-Whitney statistical test. Differences were considered significant with p < 0.05 (*). ns = non-significant. The CDG samples were obtained from PMM2-CDG, ALG11-CDG, ALG1-CDG, ALG6-CDG, MPDU1-CDG and RFT1-CDG patients, and grouped into severe, moderate and mild phenotypes. The NAFLD group is divided into liver steatosis and NASH samples.

Cohorts of 10 CDG and 12 NAFLD with varying severities of clinical symptoms were selected to assay the N-glycosylation site occupancies. The CDG samples were collected from patients with mild, moderate and severe symptoms according to the scoring system proposed by Grünewald *et al*.^30^. The CDG samples selected comprised defects in the biosynthesis upstream to the transfer of the preassembled oligosaccharide precursor to the newly synthesized protein (i.e. PMM2-CDG, ALG11-CDG, ALG1-CDG, ALG6-CDG, MPDU1-CDG, RFT1-CDG, see Table 2). These CDGs are characterized by a reduced N-glycosylation site occupancy in serum proteins such as the diagnostic marker protein transferrin. The liver disease samples were obtained from patients with NAFLD activity scores ranging from less than 3 to 6 (Table 2)^31^. Reduced N-glycosylation site occupancies were observed for the CDG samples with liver synthesized transferrin and haptoglobin, whereas no change in glycosylation frequency was observed with the glycopeptides from the immunoglobulins IgA1 and IgG2. It stands out, that under physiological conditions N-glycosylation sites are differentially glycosylated, depending on the glycoprotein. This is evident when comparing the transferrin and haptoglobin glycopeptides (Fig. 3). Both proteins are mainly synthesized in hepatocytes. Typically, transferrin contains two and haptoglobin contains four N-linked glycosylation sites. A third N-linked glycosylation site has been described at a non-canonical NXC sequon within the human transferrin at Asn491. This glycosylation site has been described to be occupied at 2 mol% in a commercially available human transferrin preparation and is not always detectable in serum protein preparations^32^,^33^. Whereas the transferrin peptide is almost completely glycosylated in healthy individuals, only 56% of the haptoglobin peptide at Asn241 is glycosylated on average. Likewise, the IgG2 peptide is completely glycosylated and the IgA1 glycopeptide is glycosylated to 85% in healthy control sera. The glycosylation sites tested to date were occupied to nearly 100% in healthy control serum samples^18^. The observation of differential degrees of glycosylation, depending on the glycoprotein is unprecedented and appears to be a tissue-independent cellular phenomenon.

### CDG and NAFLD

**Figure 4:**
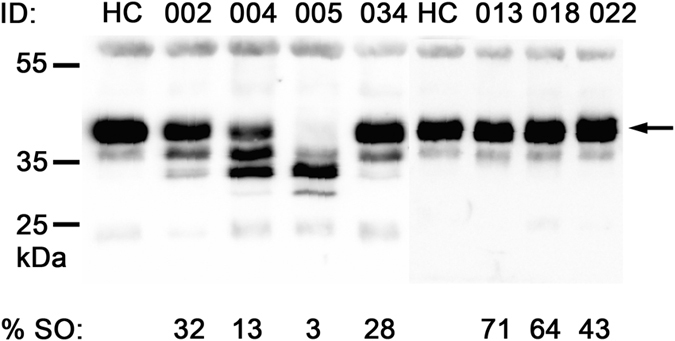
Western-blot analyses of haptoglobin from CDG and NAFLD samples. Lanes with ID 002-005 show CDG samples with increasing severity of the clinical symptoms; ID 002, PMM2-CDG, mild, ID 004, PMM2-CDG, moderate, ID 005, PMM2-CDG, severe^30^. ID 034 shows the ALG11-CDG sample and lanes with ID 013-022 show NAFLD samples with increasing clinical scores; ID 013, steatosis, score < 3, ID 018, NASH, score 4, ID 022, NASH, score 6^31^. The arrow indicates the migration position of fully glycosylated haptoglobin. The migration positions of the protein molecular weight markers are indicated at the left in kDa. The percent site occupancy of the corresponding haptoglobin glycopeptide is indicated below the lanes (% SO = percent site occupancy, ID = unique sample identifier, HC = pooled healthy control serum).

In CDG the extent of hypoglycosylation of haptoglobin can reach to as low as 3% site occupancy as seen in CDG samples with a severe clinical display (PMM2-CDG, Alg6-CDG, RFT1-CDG, Table 2), whereas for the transferrin glycosylation site the lowest value measured was 72% (RFT1-CDG, Table 2), highlighting the dynamic range of N-glycosylation site occupancies for serum glycoproteins. Reduced site occupancy for the IgA1 and IgG2 glycopeptides could not be observed in any of the CDG or NAFLD samples analyzed in this study (Fig. 3C,D). On the other hand, for the transferrin and haptoglobin glycopeptides of the NAFLD samples an increased glycosylation frequency was detected. The increase observed with the transferrin peptide corresponds to a mean value of 94 raised to 96% site occupancy (NASH group, Fig. 3A). Although the difference is minimal, a p-value of 0.026 was calculated after statistical evaluation, using the Mann-Whitney-test. An increased glycosylation within the steatosis samples was pronounced when analyzing the haptoglobin glycopeptide (Fig. 3B). The average site occupancy rose from 56 to 68%, compared to the healthy control samples. Hyperglycosylation appears to occur early during the development of liver steatosis in NAFLD. An increase in site occupancy was observed in the steatosis samples, whereas the site occupancy of the haptoglobin peptide in the NASH samples is heterogeneous, varying from mildly hypo- to hyperglycosylation. On the other hand, statistical comparison among the diseased groups did not yield significance in the difference neither between severe versus moderate or moderate versus mild CDG nor between the steatosis and NASH group (Supplemental Table 3).The variability in the NASH samples reflects the heterogeneity within this NAFLD classification. Notably, for the sample with the lowest site occupancy in the NASH group, a differential diagnosis was indicated for viral hepatitis (ID 022, Table 3). This patient with an NAS score of 6 also displayed the most advanced fibrosis stage. In contrast, patient 019 has a similar NAS score, but displayed only a mild fibrosis stage. It remains to be shown to what extent a reduced N-glycosylation site occupancy correlates with the formation of liver fibrosis. The hyperglycosylation phenotype of haptoglobin in NAFLD sera was not visible in Western blot analyses (Fig. 4, Supplemental Fig. 2). Possibly, the increase in mass due to additional N-glycosylation is not resolved during SDS-PAGE. The gain in mass of hyperglycosylated haptoglobin may be insufficient to result in a significant shift in the protein migration that can be resolved within in the mass range of haptoglobin. On the other hand, hypoglycosylation of haptoglobin is clearly resolved in CDG samples. In agreement with earlier findings, the degree of underglycosylation of haptoglobin correlates with the severity of the clinical CDG manifestations^18^ Likewise, with isoelectric focusing gel electrophoreses the CDG samples were clearly recognized, whereas the NAFLD samples were without pathological findings (Supplemental Fig. 1).

## Discussion

In this pilot study, we analyzed the N-glycosylation sites from liver derived glycoproteins and glycoproteins of lymphatic origin, using sample cohorts from CDG and NAFLD patients together with healthy control sera. Using an optimized sample preparation protocol, four glycoproteins, haptoglobin, transferrin, IgG2 and IgA1 were analyzed. Our results show that in comparison to healthy controls, haptoglobin is hyperglycosylated in liver steatosis patients, suggesting hyperglycosylation as a diagnostic marker for early stage NAFLD. Furthermore, we show that IgG2 and IgA1 of CDG patients are normally glycosylated. We found that serum glycoproteins are glycosylated to varying degrees under physiological conditions, an instance not noticed in serum proteins to date.

Increased levels of CDT are a common hallmark in ALD and CDG, and the absence of entire glycan chains in CDT has been shown^18^,^29^. Here we provide direct evidence that IgA1 and IgG2 glycosylation levels are not affected in CDG patients with a hypoglycosylation phenotype of serum transferrin and haptoglobin. Whereas for the IgG2 peptide no unglycosylated sequon could be detected, the glycosylation level of IgA1was determined to average at 82 to 90% site occupancy. The normal glycosylation levels of the immunoglobulins in CDG point to compensatory or alternative biosynthetic processes in lymphatic tissues yielding homeostatic N-glycosylation levels. Another explanation for this observation may be a rapid clearance of unglycosylated immunoglobulins in the blood via an as yet unknown processes analogous to the asialoglycoprotein receptor mediated clearance of IgA from the serum^23^. Glycosylation of the Fc-region is important for mediating the effector functions via binding to the Fc-receptors to regulate immune responses^20^. De-sialylated, galactose exposing glycoproteins are recognized by the hepatic asialoglycoprotein receptor and rapidly cleared from the blood circulation^34^. The presence of high mannose N-glycan accelerates the clearance rate of IgG through binding to the mannose receptor present on immune cells in mice^35^. Complete absence of Fc region glycosylation however results in the loss of effector functions *in vivo* as well as decreased stability and increased protein aggregation *in vitro*, which might explain the observed absence of unglycosylated IgG in the serum samples analyzed in this study^20^,^36^. Our observations that the liver derived transferrin and haptoglobin glycopeptides are hypoglycosylated, and the immunoglobulins are not affected in the CDG cases analyzed, are in good agreement with findings described by Dupre *et al*.^37^. In Western blot analyses of IgG from serum and peripheral blood mononuclear cell homogenates as well as of the glucose transporter GLUT-1 in skin fibroblast homogenate, no indications for underglycosylation in PMM2-CDG samples were detected. Based on their findings with further glycoproteins in different cell types, the authors concluded that hypoglycosylation of proteins is tissue dependent in PMM2-CDG.

Previously, we found a preferential glycosylation of the first N-linked glycosylation sites and a higher susceptibility of the second site to hypoglycosylation in transferrin and α1-antitrypsin. Hypoglycosylation was found to correlate with the severity of the clinical symptoms in CDG. Furthermore, the level of hypoglycosylation detected in transferrin of the patient samples reached as low as 41% for the most severe CDG cases^18^. Here we selected one glycosylation site per protein based on the detectability in LC-MS after proteolytic digestion with a single protease. The serum samples were prepared with minimal sample manipulations, omitting immunoaffinity purification of the proteins. CDG samples with various gene defects were selected to validate the sample preparation protocol. In this study, the lowest detected glycosylation site occupancy of the corresponding transferrin peptides was found to be 72% for equally severe diseased patients. The greater range in hypoglycosylation observed with the previous method is of advantage for assessing the severity of the clinical phenotype using transferrin as the diagnostic marker, and can be explained by an enrichment-bias towards unglycosylated transferrin epitopes induced by the immunoaffinity purification^18^. Therefore, the values obtained in this study most likely present a more precise description of the samples analyzed, since a bias in the sample preparation due to immunoaffinity enrichment was precluded. We found a dramatic dynamic range of hypoglycosylation with the haptoglobin peptide. In the healthy control samples the haptoglobin peptide is glycosylated to 56% only, a value so far not observed in proteins in non-diseased conditions. This finding is in contrast to previous reports showing a 95% N-glycosylation site occupancy for hemoglobin-affinity enriched haptoglobin^38^. It is conceivable, that the affinity enrichment introduces a bias towards glycosylated haptoglobin. Whether haptoglobin binding to free hemoglobin *in vivo* is affected by the glycosylation status of the carrier protein is not clear. The physiological significance of haptoglobin N-glycosylation site occupancies remain to be demonstrated. Nevertheless, haptoglobin appears to be more adequate for detecting changes in glycosylation frequency than other serum proteins examined to date. Haptoglobin glycosylation has been investigated in previous studies, comparing hepatocellular carcinoma patients originated from ALD, hepatitis C and hepatitis B virus infection. Quantitative analysis of purified haptoglobin N-glycans indicated an increase in bi-fucosylated glycans in hepatocellular carcinoma patients, providing a potential use as a marker for the detection of early stage hepatocellular carcinoma patients^39^. In these studies however, the glycosylation site occupancy has not been addressed. A comprehensive assessment of the haptoglobin glycosylation status from differing liver disease conditions might be valuable for identifying predictive liver disease markers in future studies.

The N-glycosylation site occupancy of serum proteins has not been determined in NAFLD patients in previous studies. Originally, reduced glycosylation was observed in ALD and the determination of CDT levels are used as a reliable means to monitor disease progression and patient compliance^28^. The cytochrome CytP2E1 is upregulated in ALD and NASH, and made responsible for the increased production of reactive oxygen species in the corresponding tissue^40^. In this regard it is surprising to find opposing glycosylation levels in NAFLD and ALD patients, indicating that the regulation of N-glycosylation site occupancy is independent of CytP2E1 mediated oxidative stress. In search for diagnostic markers to differentiate alcoholic hepatitis from NASH, Ohtsuka *et al*.^8^ found increasing levels of CDT in alcoholic hepatitis patients in comparison to patients with NASH. The relationship of patient to healthy control samples, however has not been investigated, leaving questions about the levels of CDT in NASH unanswered. Alterations in the N-glycosylation site occupancy are a result of interferences in the biosynthesis upstream to the transfer of glycan precursors to the newly synthesized polypeptide in the ER, whereas alterations in the N-glycans structures can be attributed to interferences in the glycan maturation and processing in the Golgi. Deficient glycan processing enzymes or defective Golgi associated proteins can be involved in this instance. Here we determined a clear hyperglycosylation of the haptoglobin peptide in sera obtained from liver steatosis patients. The hyperglycosylation of haptoglobin occurred independent of liver inflammation, prior to the development of NASH in the early staged NAFLD patients. The observed occurrence of hyperglycosylation in NAFLD might be a result of interferences early in the biosynthesis of the N-glycans. On the other hand, increases in site occupancy will most likely affect the N-glycan maturation, due to an increased load of substrate for the Golgi localized glycan processing enzymes. For instance, the undergalactosylation of serum N-glycans was proposed as a glycomarker for liver inflammation and NASH-related fibrosis^11^,^12^. In subsequent studies, increased fucosylation of haptoglobin N-glycans as a diagnostic marker for NASH were suggested^41^,^42^. In this context, analyzing the glycosylation site occupancy provides a sensitive and specific means to diagnose liver steatosis early in NAFLD before the development of NASH.

In conclusion our data suggest that under physiological conditions N-glycosylation sites can be occupied with different frequencies, depending on the glycoprotein, an instance as yet not described in serum proteins. For the glycoproteins investigated, glycosylation site occupancies were also dependent on the tissue of origin. Furthermore, no changes to healthy controls were observed with the immunoglobulin derived peptides in the CDG patients. Our third finding that in NAFLD haptoglobin is hyperglycosylated opens new opportunities in biomarker research for fatty liver diseases.

## Methods

### Serum Samples

Ten CDG samples obtained from patients with confirmed diagnosis of PMM2-CDG, Alg11CDG, Alg1-CDG, Alg6-CDG, MPDU1-CDG and RFT1-CDG and 5 healthy control samples were used. Twelve NAFLD samples, 4 with confirmed diagnosis of steatosis and 8 samples with confirmed diagnosis of NASH were evaluated. The study protocol was approved by the Ethics Committee of the University Hospital Zurich and all NAFLD patients were provided with written, informed consent. All experiments were performed in accordance with the guidelines and regulations of the Ethics Committee of the University Hospital Zurich.

### Preparation of serum samples

One μl of serum was diluted 20-fold to give 20 μl of 50 mM triethylammonium bicarbonate buffer (Sigma, Switzerland), pH 8.5, 0.1% (w/v) SDS, 5 mM tris(2-carboxyethyl)phosphine (ThermoFischer, USA) and incubated for one hour at 60 °C. The samples were cooled to room temperature, 10 μl of freshly prepared 50 mM iodoacetamide were added and the samples were incubated at room temperature in the dark. After 40 min, a five-fold excess of dithiothreitol over iodoacetamide was added to quench the reaction and the samples were digested with trypsin for 16 h at 37 °C (10 μl of 0.5 μg trypsin/μl in H_2_O, sequencing grade, Roche, Switzerland). The samples were acidified with 4 μl of 10% (v/v) acetic acid and stored at −20 °C until further processing. A 0.5 ml Dowex AG50 × 8 column was pre-cycled with 5% (v/v) ammonia in 25% (v/v) acetonitrile and equilibrated with 0.8% (v/v) acetic acid in 25% (v/v) acetonitrile. The samples were diluted to 8 ml with 0.8% (v/v) acetic acid, 25% (v/v) acetonitrile, loaded onto the column, washed with 6 ml of 0.8% (v/v) acetic acid, 25% (v/v) acetonitrile and eluted with 2 ml 5% (v/v) ammonia, 25% (v/v) acetonitrile. The volumes of the eluates were concentrated by rotary evaporation before lyophilization. The dried samples were re-dissolved in 44 μl 20 mM ammonium bicarbonate buffer prepared with ^18^O-H_2_O (>97% ^18^O, Cambridge Isotope Laboratories, USA) and 6 units of N-glycosidase F (Roche, Switzerland) in 20 mM ^18^O-H_2_O ammonium bicarbonate buffer were added. The peptides were de-glycosylated for 6 h at 37 °C and aliquots were desalted with ZipTip C18 tips (Millipore, USA) prior submission to mass spectrometry.

A standard peptide mix consisting of synthetic peptides with c-terminal labeled arginine or lysine corresponding to the target glycopeptide sequences with asparagine or aspartic acid at the glycosylation sites was prepared (>70% purity, Intavis AG, Germany, Table 1, Supplemental Fig. 3). The peptides were dissolved in 2% (v/v) acetonitrile, 0.1% (v/v) formic acid at a concentration of 100 or 50 fmol per μl for the transferrin, IgG2 and IgA1 peptides and 10 or 5 fmol per μl for the haptoglobin peptides. An aliquot corresponding to 4% of the sample was desalted with ZipTip C18 tips, dried by rotary evaporation, and re-dissolved in 10 μl of the standard peptide mix. For each sample, 4 μl were injected onto a nano-frit column (15 cm × 75 μm; OD 375 μm; beads, Magic C18 AQ, 3 μm, 200 Å; Bischoff Chromatography, Germany), connected to a spray tip (PicoTip emitter, New Objective, USA), coupled to an Eksigent nano LC system (Eksigent, AB Sciex, USA). The column was kept at 50 °C. Peptides were eluted using a gradient from 3 to 45% acetonitrile, 0.1% (v/v) formic acid in water for 40 min, and a flow rate of 300 nl/min.

### LC-MS-MS peptide sequencing

Glycosylation site containing peptides were identified by LC-MS-MS peptide sequencing. The dried peptides were re-suspended in 3% (v/v) acetonitrile and 0.1% (v/v) formic acid and analyzed on a LTQ-Orbitrap Velos mass spectrometer (ThermoFischer Scientific, USA) coupled to an Eksigent nano LC system (Eksigent, AB Sciex, USA). The analyses were performed in the data-dependent acquisition mode. The ten most intense precursor ions in the mass range m/z 300–2000 were selected for HCD spectrum acquisition with a normalized collision energy of 40 V. Single charged precursors and unassigned charge states were rejected and the minimal signal intensity required for MS2 was 2000 counts. Dynamic exclusion was switched on with an exclusion list size of 50, and an exclusion mass width relative to mass of ±10 ppm. Values were excluded from MS/MS for 45 s. The instrument was calibrated externally according to the manufacturer’s instructions. From the LC-MS-MS peptide sequencing results of a healthy control serum, treated with PNGase F in ^18^O-H_2_O buffer, a spectral library was generated and imported into Skyline for the MRM-method development. The glycopeptides were selected on the basis of robust detection after identification using the Mascot search engine (Matrix Science, UK; Version: 2.4.1.). Mascot was set up to search the human protein entries of the Swissprot database (downloaded 04.12.2012, 170103 entries, containing decoy dataset and common contaminants) assuming the digestion enzyme trypsin with maximal one missed cleavage. Mascot was searched with a fragment ion mass tolerance of 0.60 Da and a parent ion tolerance of 10.0 ppm. Carbamidomethyl-alkylation of cysteine was specified as fixed modifications. Oxidation of Met, Gln conversion to pyro-Glu and ^18^O-incorporation in Asn residues were specified in Mascot as variable modifications. Suitable tryptic peptides were selected on the basis of high peptide identification scores for the synthesis of isotopically labeled standards. Selected peptides should not contain missed cleavage sites or methionine whenever possible. The peptide length was kept short with maximal one missed tryptic cleavage site to enable reproducible and unambiguous identifications and chromatographic separation of deaminated and non-deaminated peptide variants.

### MRM-method development

Four glycosylation site containing peptides were selected from four proteins, based on robust detection in LC-MS-MS peptide sequencing. Theoretical MRM transitions were computed using the Skyline software environment, and six transition per precursor were analyzed^43^. LC-MRM-MS files were imported and precursor-fragment transitions were manually inspected for correct assignment and accurate peak integration. Precursor peaks were identified by co-elution of transitions derived from the isotopically labeled standard peptides included in the samples. The four transitions showing most intense signals and highest selectivity for each precursor in comparison to the spectral library were selected. Whenever possible, transitions including the glycosylation site in the fragment ion were selected. For the four selected peptides a list was assembled of 104 transitions (Supplemental Table 1). The isotopically labeled standard peptide mix was used to validate the transitions and to empirically optimize collisional energies (Table 1).

The analyses were performed in positive ion mode on a QTRAP 5500 mass spectrometer (AB Sciex, USA) equipped with a nanospray ion source. The interface temperature was set to 170 °C, an ion spray voltage from 2000–2500 V, the ion source gas pressure was 6–10 psi, the curtain gas pressure was 25 psi, and the collision gas set to medium was used. The declustering potential had a value of 80 V, collision cell exit potential of 13 V, and entrance potential of 10 V. Further settings were unit resolution for both Q1 and Q3 and 3 ms pause between mass ranges. The total scan time was 2.6 s.

For quantitative calculations of peptide abundances only transitions unique to the target peptide were used (Table 1). The product peak areas were validated manually and assessed for the co-elution of the sample peptides with the isotope labeled standard peptides added to the samples. Molar relative response factors (MRRF) were computed for spontaneously deaminated peptides (i.e. deamination-^16^O, not glycosylated, MRRF-^16^O), enzymatically deaminated peptides (i.e. deamination-^18^O, deglycosylated, MRRF-^18^O) and non-deaminated peptides (i.e. not glycosylated, MRRF-N):


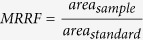


The relative glycosylation site occupancy was calculated using the MRRF:


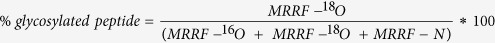


The portion of spontaneously deaminated peptides was calculates as follows.


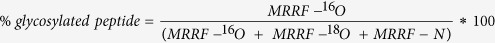


### Western blot analysis of serum haptoglobin

Serum samples were diluted 1:250 in H_2_O and serum protein precipitated by the addition of 1/10 volume 100% (w/v) trichloroacetic acid (w/vol). Precipitated protein was centrifuged, protein pellets washed with ice-cold acetone and dried at 95 °C for 10 min. For SDS-PAGE, dried pellets were dissolved in 200 μl 2x Laemmli Sample Buffer containing 5% (v/v) 2-mercaptoethanol and boiled for 5 min at 95 °C. Proteins were separated on a 10% (w/v) SDS polyacrylamide gel and blotted onto Protran BA85 nitrocellulose membrane (Whatman) and blocked at room temperature for 1 h in PBS-0.1% (v/v) Tween −5% (w/v) non-fat dry milk (PBSTP). The membrane was incubated for 1 h at room temperature with 1/3500 rabbit-α-human haptoglobin (DakoCytomation), followed by incubation with 1/5000 goat-α-rabbit –HRPO (Santa Cruz). All incubation steps were performed in PBSTP and the membrane was subsequently washed three times in PBS −0.1% Tween. Detection occurred using ECL (GE Healthcare Life Sciences).

## Additional Information

**How to cite this article**: Hülsmeier, A. J. *et al*. Glycosylation site occupancy in health, congenital disorder of glycosylation and fatty liver disease. *Sci. Rep*. **6**, 33927; doi: 10.1038/srep33927 (2016).

## Supplementary Material

Supplementary Information

Supplementary Data

## Figures and Tables

**Table 1:** Peptides used to determine the N-glycosylation site occupancies.

Protein	Peptide	m/z	Charge	Fragments				av. RT (min)	av. dot p	Description
sp|P02787|TRFE_HUMAN	K.ILRQQQHLFGSNVTDCSGNFCLFR.S	727.6059	4	y_4_	b_13_^2+^	b_14_^2+^	b_15_^2+^	36.7	0.81	standard-N
	K.ILRQQQHLFGS**N**VTD**C**SGNF**C**LF**R**.S	727.8519	4	y_4_	b_13_^2+^	b_14_^2+^	b_15_^2+^	37.1	0.80	standard-D
	K.ILRQQQHLFGS**N**VTD**C**SGNF**C**LFR.S	725.3498	4	y_4_	b_13_^2+^	b_14_^2+^	b_15_^2+^	37.1	0.76	deamination-16O
	K.ILRQQQHLFGS**N**VTD**C**SGNF**C**LFR.S	725.8509	4	y_4_	b_13_^2+^	b_14_^2+^	b_15_^2+^	37.1	0.81	deamination-18O
	K.ILRQQQHLFGSNVTD**C**SGNF**C**LFR.S	725.1038	4	y_4_	b_13_^2+^	b_14_^2+^	b_15_^2+^	36.7	0.80	no deamination
										
sp|P00738|HPT_HUMAN	K.VVLHPNYSQVDIGLI**K**.L	601.6800	3	y_6_	y_14_^2+^	b_9_	b_10_^2+^	36.1	0.73	standard-N
	K.VVLHP**N**YSQVDIGLI**K**.L	602.0080	3	y_6_	y_14_^2+^	b_9_	b_10_^2+^	36.5	0.72	standard-D
	K.VVLHP**N**YSQVDIGLIK.L	599.3366	3	y_6_	y_14_^2+^	b_9_	b_10_^2+^	36.5	0.67	deamination-16O
	K.VVLHP**N**YSQVDIGLIK.L	600.0047	3	y_6_	y_14_^2+^	b_9_	b_10_^2+^	36.5	0.88	deamination-18O
	K.VVLHPNYSQVDIGLIK.L	599.0086	3	y_6_	y_14_^2+^	b_9_	b_10_^2+^	36.1	0.73	no deamination
										
sp|P01876|IGHA1_HUMAN	R.LSLHRPALEDLLLGSEANLT**C**TLTGL**R**.D	744.1571	4	y_10_	y_11_	b_11_	b_12_^2+^	43.4	0.74	standard-N
	R.LSLHRPALEDLLLGSEA**N**LT**C**TLTGL**R**.D	744.4031	4	y_10_	y_11_	b_11_	b_12_^2+^	44.2	0.76	standard-D
	R.LSLHRPALEDLLLGSEA**N**LT**C**TLTGLR.D	741.9010	4	y_10_	y_11_	b_11_	b_12_^2+^	44.2	0.63	deamination-16O
	R.LSLHRPALEDLLLGSEA**N**LT**C**TLTGLR.D	742.4021	4	y_10_	y_11_	b_11_	b_12_^2+^	44.2	0.71	deamination-18O
	R.LSLHRPALEDLLLGSEANLT**C**TLTGLR.D	741.6550	4	y_10_	y_11_	b_11_	b_12_^2+^	43.4	0.70	no deamination
										
sp|P01859|IGHG2_HUMAN	K.TKPREEQFNSTF**R**.V	825.4171	2	y_4_	y_7_	y_11_^2+^	b_9_	28.0	0.75	standard-N
	K.TKPREEQF**N**STF**R**.V	825.9091	2	y_4_	y_7_	y_11_^2+^	b_9_	28.6	0.81	standard-D
	K.TKPREEQF**N**STFR.V	820.9050	2	y_4_	y_7_	y_11_^2+^	b_9_	28.6	na	deamination-16O
	K.TKPREEQF**N**STFR.V	821.9071	2	y_4_	y_7_	y_11_^2+^	b_9_	28.6	0.86	deamination-18O
	K.TKPREEQFNSTFR.V	820.4130	2	y_4_	y_7_	y_11_^2+^	b_9_	28.0	na	no deamination

The areas of the MRM transitions corresponding to the fragment ions b_13_^2+^, b_14_^2+^, b_15_^2+^ of the transferrin peptide, the y_14_^2+^, b_9_, b_10_^2+^ of haptoglobin peptide, the y_7_, b_18_^2+^ of the IgA_1_ peptide and the y_11_^2+^, y_7_, b_9_ of the IgG_2_ peptide were used for semi-quantitative computation of the N-glycosylation site occupancies. The deaminated asparagine residues of the N-glycosylation sequons are marked with **N**. The stable isotope labeled lysine and arginine residues of the standard peptides are labeled with **K** and **R**, respectively. **C** denotes carbamidomethylated cysteine residues. The synthetic peptides denoted with standard-N or standard-D were added to the sampled prior MRM-MS analyses. The Swiss-Prot designations for the proteins are shown. The dotp values are the similarity measures between product peak areas and the corresponding intensities in the spectral library used in Skyline. The average dotp values were calculated from manually validated MRM transitions of the peptides. The dotp values for all peptides investigated are listed in Supplemental data dotp values.xlsx. “na” = not applicable, for those peptides no product peak areas could be validated.

**Table 2:** N-glycosylation site occupancies in health and liver disease conditions.

ID	Sample	OMIM entry	Clinical score	TRFE		HPT		IGHA1		IGHG2	
				mean	SEM	mean	SEM	mean	SEM	mean	SEM
001	PMM2-CDG	601785	*	86	2.8	3	1.0	82	2.8	100	0.0
002	PMM2-CDG	601785	mild	90	2.1	32	4.6	83	1.0	100	0.0
003	PMM2-CDG	601785	mild/moderate	91	0.7	24	3.1	86	1.0	100	0.0
004	PMM2-CDG	601785	moderate	91	1.0	13	2.2	87	0.2	100	0.0
005	PMM2-CDG	601785	severe	84	1.8	3	0.5	86	2.1	100	0.0
034	AlG11-CDG	613661	*	94	0.1	28	1.2	90	2.7	100	0.0
036	AlLG1-CDG	608540	moderate	82	2.1	7	0.4	91	0.1	100	0.0
037	ALG6-CDG	603147	severe	85	0.5	3	0.3	90	0.5	100	0.0
038	MPDU1-CDG	604041	moderate/severe	91	0.5	12	3.4	87	1.7	100	0.0
039	RFT1-CDG	612015	severe	72	1.7	3	0.7	91	1.8	100	0.0
007	healthy	na	na	93	0.9	61	2.5	84	1.8	100	0.0
008	healthy	na	na	94	0.5	57	3.2	86	1.6	100	0.0
009	healthy	na	na	93	1.1	54	1.8	85	1.3	100	0.0
010	healthy	na	na	95	0.5	52	3.1	84	1.3	100	0.0
011	healthy	na	na	95	0.5	56	6.5	86	4.3	100	0.0
012	Steatosis	nd	<3	95	1.4	73	4.0	86	1.9	100	0.0
013	Steatosis	nd	<3	94	0.5	71	5.6	85	1.0	100	0.0
015	Steatosis	nd	<3	95	0.7	61	5.8	84	4.0	100	0.0
016	Steatosis	nd	<3	95	0.5	68	1.9	87	1.4	100	0.0
014	NASH	nd	5	95	0.2	69	1.9	86	1.5	100	0.0
017	NASH	nd	3	95	0.8	59	5.2	86	0.9	100	0.0
018	NASH	nd	4	96	1.0	64	1.4	86	1.9	100	0.0
019	NASH	nd	5–6	95	0.7	69	4.1	82	1.4	100	0.0
020	NASH	nd	4	95	1.5	60	12.3	88	2.6	100	0.0
021	NASH	nd	4	94	0.7	53	4.4	88	3.3	100	0.0
022	NASH	nd	6	95	1.3	43	3.9	91	1.5	100	0.0
023	NASH	nd	*	94	0.7	58	11.5	90	2.0	100	0.0

The values for each glycosylation site are averaged from three technical replicates, starting with the serum sample preparation. SEM = standard error of mean. Average values derived from 5 samples from healthy serum, 4 liver steatosis samples and 8 NASH samples. OMIM = Online Mendelian Inheritance in Man, na = not applicable, nd = not determined, ID = unique sample identifier, TRFE = transferrin, HPT = haptoglobin, IGHA1 = human IgA1, IGHG2 = human IgG2, (*) = no clinical information available. The clinical score values are displayed according to Grünewald *et al*. or Kleiner *et al*. (NAFLD activity scores), respectively^30^,^31^.

**Table 3:** NAFLD clinical data.

ID	Sample	Clinical score	Age	Gender	Steatosis level	Hepatocyte ballooning	Inflammation	Fibrose stage	Differential diagnosis
012	Steatosis	<3	53	f	mixed vesicular	isolated ballooning	borderline to steatohepatitis	Fibrose stage 2	
013	Steatosis	<3	40	m	mixed vesicular	no ballooning	some portal inflammation	none	
015	Steatosis	<3	34	m	mixed vesicular	no ballooning reported	none	focal pericellular fibrosis	
016	Steatosis	<3	40	f	mainly micovesicular	no ballooning	none	minor pericellular fibrosis	nutritional or medicinal intoxication
014	NASH	5	40	m	mixed vesicular	ballooning	minor lobular inflammation	isolated minor pericellular fibrosis	
017	NASH	3	52	m	moderate mixed vesicular	isolated ballooning	no lobular or portal inflammation	no portal or pericellular fibrosis	
018	NASH	4	50	f	moderate mainly macrovesicular	discreet ballooning	sparse mixed cellular inflammarory portal infiltration	discreet pericentral fibrosis and perisinusoidal collagen deposition	
019	NASH	5–6	54	f	mainly macrovesicular	ballooning	isolated, sparse portal inflammatory infiltration	discreet focal pericellular fibrosis	
020	NASH	4	54	f	mixed vesicular	pronounced ballooning	focal necro-inflammatory activity	perisinusoidal, portal and periportal fibrosis, focal septum formation	
021	NASH	4	31	m	mixed vesicular	isolated ballooning	no lobular or portal inflammation	minor pericellular fibrosis	
022	NASH	6	58	m	mixed vesicular	ballooning	portal and lobular inflammation	septal and portal fibrosis	additional viral hepatitis appears to be possible
023	NASH	*	66	f	mainly macrovesicular	ballooning	no definite florid lobular inflammation	minor focal pericellular fibrosis	

ID = unique sample identifier, the clinical score values are displayed as NAFLD activity scores^31^.
