# Supplementary material for: Glycosylation site occupancy in health, congenital disorder of glycosylation and fatty liver disease
Source: Sci Rep. 2016 Oct 11;6:33927. doi: 10.1038/srep33927 (PMC5057071; doi:10.1038/srep33927)
Supplement: Supplementary Information [file srep33927-s1.pdf]

Glycosylation site occupancy in health, congenital disorder of glycosylation and fatty liver disease

Andreas J. Hülsmeier‡§, Micha Tobler¶, Patricie Burda¶ and Thierry Hennet‡

From the ‡Institute of Physiology, University of Zürich, Winterthurerstrasse 190, 8057 Zürich, Switzerland and ¶Division of Metabolism and Molecular Pediatrics, University Children's Hospital Zürich, Steinwiesstrasse 75, 8032 Zürich, Switzerland

§To whom correspondence should be addressed. Tel.: 41-44-635-5104; Fax: 41-44-635-6814; E-mail: *andreas.huelsmeier@uzh.ch*

Supplemental Table 1 MRM transitions

Supplemental Table 2 ^{16}O deamination reactions

Supplemental Figure 1. Isoelectric focusing gel electrophoresis of transferrin of the samples analyzed.

Supplemental Figure 2. Western-blot analyses of haptoglobin of the samples analyzed.

Supplemental Table 3 Statistical comparison of the CDG and NAFLD disease groups.

Supplemental Figure 3. MALDI-TOF-MS of the standard peptide mix.

Supplemental Table 1 MRM transitions

Q1	Q3	dwell time (ms)	description	CE (V)
725.850866	595.302078	15	sp|P02787|TRFE_HUMAN.ILRQQQHLFGSN[DeW]VTDC[CAM]SGNFC[CAM]LFR.+4y4.light	31
725.850866	762.912004	15	sp|P02787|TRFE_HUMAN.ILRQQQHLFGSN[DeW]VTDC[CAM]SGNFC[CAM]LFR.+4b13+2.light	31
725.850866	813.435843	15	sp|P02787|TRFE_HUMAN.ILRQQQHLFGSN[DeW]VTDC[CAM]SGNFC[CAM]LFR.+4b14+2.light	31
725.850866	870.949315	15	sp|P02787|TRFE_HUMAN.ILRQQQHLFGSN[DeW]VTDC[CAM]SGNFC[CAM]LFR.+4b15+2.light	31
725.103801	595.302078	15	sp|P02787|TRFE_HUMAN.ILRQQQHLFGSNVTDC[CAM]SGNFC[CAM]LFR.+4y4.light	31
725.103801	761.417874	15	sp|P02787|TRFE_HUMAN.ILRQQQHLFGSNVTDC[CAM]SGNFC[CAM]LFR.+4b13+2.light	31
725.103801	811.941713	15	sp|P02787|TRFE_HUMAN.ILRQQQHLFGSNVTDC[CAM]SGNFC[CAM]LFR.+4b14+2.light	31
725.103801	869.455184	15	sp|P02787|TRFE_HUMAN.ILRQQQHLFGSNVTDC[CAM]SGNFC[CAM]LFR.+4b15+2.light	31
727.605868	605.310347	15	sp|P02787|TRFE_HUMAN.ILRQQQHLFGSNVTDC[CAM]SGNFC[CAM]LFR.+4y4.heavy	31
727.605868	761.417874	15	sp|P02787|TRFE_HUMAN.ILRQQQHLFGSNVTDC[CAM]SGNFC[CAM]LFR.+4b13+2.heavy	31
727.605868	811.941713	15	sp|P02787|TRFE_HUMAN.ILRQQQHLFGSNVTDC[CAM]SGNFC[CAM]LFR.+4b14+2.heavy	31
727.605868	869.455184	15	sp|P02787|TRFE_HUMAN.ILRQQQHLFGSNVTDC[CAM]SGNFC[CAM]LFR.+4b15+2.heavy	31
966.797315	1160.49756	15	sp|P02787|TRFE_HUMAN.ILRQQQHLFGSN[+1]VTDC[CAM]SGNFC[CAM]LFR.+3y9.light	44
966.797315	712.375675	15	sp|P02787|TRFE_HUMAN.ILRQQQHLFGSN[+1]VTDC[CAM]SGNFC[CAM]LFR.+3b12+2.light	44
966.797315	761.909882	15	sp|P02787|TRFE_HUMAN.ILRQQQHLFGSN[+1]VTDC[CAM]SGNFC[CAM]LFR.+3b13+2.light	44
966.797315	869.947192	15	sp|P02787|TRFE_HUMAN.ILRQQQHLFGSN[+1]VTDC[CAM]SGNFC[CAM]LFR.+3b15+2.light	44
725.349805	595.302078	15	sp|P02787|TRFE_HUMAN.ILRQQQHLFGSN[+1]VTDC[CAM]SGNFC[CAM]LFR.+4y4.light	31
725.349805	761.909882	15	sp|P02787|TRFE_HUMAN.ILRQQQHLFGSN[+1]VTDC[CAM]SGNFC[CAM]LFR.+4b13+2.light	31
725.349805	812.433721	15	sp|P02787|TRFE_HUMAN.ILRQQQHLFGSN[+1]VTDC[CAM]SGNFC[CAM]LFR.+4b14+2.light	31
725.349805	869.947192	15	sp|P02787|TRFE_HUMAN.ILRQQQHLFGSN[+1]VTDC[CAM]SGNFC[CAM]LFR.+4b15+2.light	31
727.851872	605.310347	15	sp|P02787|TRFE_HUMAN.ILRQQQHLFGSN[+1]VTDC[CAM]SGNFC[CAM]LFR.+4y4.heavy	31
727.851872	761.909882	15	sp|P02787|TRFE_HUMAN.ILRQQQHLFGSN[+1]VTDC[CAM]SGNFC[CAM]LFR.+4b13+2.heavy	31
727.851872	812.433721	15	sp|P02787|TRFE_HUMAN.ILRQQQHLFGSN[+1]VTDC[CAM]SGNFC[CAM]LFR.+4b14+2.heavy	31
727.851872	869.947192	15	sp|P02787|TRFE_HUMAN.ILRQQQHLFGSN[+1]VTDC[	31

			CAM]SGNFC[CAM]LFR.+4b15+2.heavy	
821.907116	902.425253	15	sp|P01859|IGHG2_HUMAN.TKPREEQFN[DeW]STFR.+2y7.light	48
821.907116	510.267073	15	sp|P01859|IGHG2_HUMAN.TKPREEQFN[DeW]STFR.+2y4.light	48
821.907116	707.335795	15	sp|P01859|IGHG2_HUMAN.TKPREEQFN[DeW]STFR.+2y11+2.light	48
821.907116	1133.547159	15	sp|P01859|IGHG2_HUMAN.TKPREEQFN[DeW]STFR.+2b9.light	48
820.412985	899.436992	15	sp|P01859|IGHG2_HUMAN.TKPREEQFNSTFR.+2y7.light	48
820.412985	510.267073	15	sp|P01859|IGHG2_HUMAN.TKPREEQFNSTFR.+2y4.light	48
820.412985	705.841664	15	sp|P01859|IGHG2_HUMAN.TKPREEQFNSTFR.+2y11+2.light	48
820.412985	1130.558898	15	sp|P01859|IGHG2_HUMAN.TKPREEQFNSTFR.+2b9.light	48
825.41712	909.445261	15	sp|P01859|IGHG2_HUMAN.TKPREEQFNSTFR.+2y7.heavy	48
825.41712	520.275342	15	sp|P01859|IGHG2_HUMAN.TKPREEQFNSTFR.+2y4.heavy	48
825.41712	710.845799	15	sp|P01859|IGHG2_HUMAN.TKPREEQFNSTFR.+2y11+2.heavy	48
825.41712	1130.558898	15	sp|P01859|IGHG2_HUMAN.TKPREEQFNSTFR.+2b9.heavy	48
820.904993	900.421008	15	sp|P01859|IGHG2_HUMAN.TKPREEQFN[+1]STFR.+2y7.light	48
820.904993	510.267073	15	sp|P01859|IGHG2_HUMAN.TKPREEQFN[+1]STFR.+2y4.light	48
820.904993	706.333672	15	sp|P01859|IGHG2_HUMAN.TKPREEQFN[+1]STFR.+2y11+2.light	48
820.904993	1131.542914	15	sp|P01859|IGHG2_HUMAN.TKPREEQFN[+1]STFR.+2b9.light	48
825.909128	910.429277	15	sp|P01859|IGHG2_HUMAN.TKPREEQFN[+1]STFR.+2y7.heavy	48
825.909128	520.275342	15	sp|P01859|IGHG2_HUMAN.TKPREEQFN[+1]STFR.+2y4.heavy	48
825.909128	711.337807	15	sp|P01859|IGHG2_HUMAN.TKPREEQFN[+1]STFR.+2y11+2.heavy	48
825.909128	1131.542914	15	sp|P01859|IGHG2_HUMAN.TKPREEQFN[+1]STFR.+2b9.heavy	48
989.533693	1222.634594	15	sp|P01876|IGHA1_HUMAN.LSLHRPALEDLLLGSEAN[DeW]LTC[CAM]TLTGLR.+3y11.light	45
989.533693	1151.59748	15	sp|P01876|IGHA1_HUMAN.LSLHRPALEDLLLGSEAN[DeW]LTC[CAM]TLTGLR.+3y10.light	45
989.533693	1245.694997	15	sp|P01876|IGHA1_HUMAN.LSLHRPALEDLLLGSEAN[DeW]LTC[CAM]TLTGLR.+3b11.light	45
989.533693	679.893169	15	sp|P01876|IGHA1_HUMAN.LSLHRPALEDLLLGSEAN[DeW]LTC[CAM]TLTGLR.+3b12+2.light	45
742.402089	820.434549	15	sp|P01876|IGHA1_HUMAN.LSLHRPALEDLLLGSEAN[DeW]LTC[CAM]TLTGLR.+4y7.light	32
742.402089	888.541397	15	sp|P01876|IGHA1_HUMAN.LSLHRPALEDLLLGSEAN[DeW]LTC[CAM]TLTGLR.+4b8.light	32

742.402089	679.893169	15	sp|P01876|IGHA1_HUMAN.LSLHRPALEDLLLGSEAN[DeW]LTC[CAM]TLTGLR.+4b12+2.light	32
742.402089	967.017394	15	sp|P01876|IGHA1_HUMAN.LSLHRPALEDLLLGSEAN[DeW]LTC[CAM]TLTGLR.+4b18+2.light	32
988.537606	1219.646333	15	sp|P01876|IGHA1_HUMAN.LSLHRPALEDLLLGSEANLTC[CAM]TLTGLR.+3y11.light	45
988.537606	1148.609219	15	sp|P01876|IGHA1_HUMAN.LSLHRPALEDLLLGSEANLTC[CAM]TLTGLR.+3y10.light	45
988.537606	1245.694997	15	sp|P01876|IGHA1_HUMAN.LSLHRPALEDLLLGSEANLTC[CAM]TLTGLR.+3b11.light	45
988.537606	679.893169	15	sp|P01876|IGHA1_HUMAN.LSLHRPALEDLLLGSEANLTC[CAM]TLTGLR.+3b12+2.light	45
991.873696	1229.654602	15	sp|P01876|IGHA1_HUMAN.LSLHRPALEDLLLGSEANLTC[CAM]TLTGLR.+3y11.heavy	45
991.873696	1158.617488	15	sp|P01876|IGHA1_HUMAN.LSLHRPALEDLLLGSEANLTC[CAM]TLTGLR.+3y10.heavy	45
991.873696	1245.694997	15	sp|P01876|IGHA1_HUMAN.LSLHRPALEDLLLGSEANLTC[CAM]TLTGLR.+3b11.heavy	45
991.873696	679.893169	15	sp|P01876|IGHA1_HUMAN.LSLHRPALEDLLLGSEANLTC[CAM]TLTGLR.+3b12+2.heavy	45
741.655024	820.434549	15	sp|P01876|IGHA1_HUMAN.LSLHRPALEDLLLGSEANLTC[CAM]TLTGLR.+4y7.light	32
741.655024	888.541397	15	sp|P01876|IGHA1_HUMAN.LSLHRPALEDLLLGSEANLTC[CAM]TLTGLR.+4b8.light	32
741.655024	679.893169	15	sp|P01876|IGHA1_HUMAN.LSLHRPALEDLLLGSEANLTC[CAM]TLTGLR.+4b12+2.light	32
741.655024	965.523264	15	sp|P01876|IGHA1_HUMAN.LSLHRPALEDLLLGSEANLTC[CAM]TLTGLR.+4b18+2.light	32
744.157091	830.442818	15	sp|P01876|IGHA1_HUMAN.LSLHRPALEDLLLGSEANLTC[CAM]TLTGLR.+4y7.heavy	32
744.157091	888.541397	15	sp|P01876|IGHA1_HUMAN.LSLHRPALEDLLLGSEANLTC[CAM]TLTGLR.+4b8.heavy	32
744.157091	679.893169	15	sp|P01876|IGHA1_HUMAN.LSLHRPALEDLLLGSEANLTC[CAM]TLTGLR.+4b12+2.heavy	32
744.157091	965.523264	15	sp|P01876|IGHA1_HUMAN.LSLHRPALEDLLLGSEANLTC[CAM]TLTGLR.+4b18+2.heavy	32
988.865612	1220.630349	15	sp|P01876|IGHA1_HUMAN.LSLHRPALEDLLLGSEAN[+1]LTC[CAM]TLTGLR.+3y11.light	45
988.865612	1149.593235	15	sp|P01876|IGHA1_HUMAN.LSLHRPALEDLLLGSEAN[+1]LTC[CAM]TLTGLR.+3y10.light	45
988.865612	1245.694997	15	sp|P01876|IGHA1_HUMAN.LSLHRPALEDLLLGSEAN[+1]LTC[CAM]TLTGLR.+3b11.light	45
988.865612	679.893169	15	sp|P01876|IGHA1_HUMAN.LSLHRPALEDLLLGSEAN[+1]LTC[CAM]TLTGLR.+3b12+2.light	45
992.201701	1230.638618	15	sp|P01876|IGHA1_HUMAN.LSLHRPALEDLLLGSEAN[+1]LTC[CAM]TLTGLR.+3y11.heavy	45
992.201701	1159.601504	15	sp|P01876|IGHA1_HUMAN.LSLHRPALEDLLLGSEAN[+1]LTC[CAM]TLTGLR.+3y10.heavy	45
992.201701	1245.694997	15	sp|P01876|IGHA1_HUMAN.LSLHRPALEDLLLGSEAN[+1]LTC[CAM]TLTGLR.+3b11.heavy	45
992.201701	679.893169	15	sp|P01876|IGHA1_HUMAN.LSLHRPALEDLLLGSEAN[+1]LTC[CAM]TLTGLR.+3b12+2.heavy	45
741.901028	820.434549	15	sp|P01876|IGHA1_HUMAN.LSLHRPALEDLLLGSEAN[+	32

			1]LTC[CAM]TLTGLR.+4y7.light	
741.901028	888.541397	15	sp|P01876|IGHA1_HUMAN.LSLHRPALEDLLLGSEAN[+1]LTC[CAM]TLTGLR.+4b8.light	32
741.901028	679.893169	15	sp|P01876|IGHA1_HUMAN.LSLHRPALEDLLLGSEAN[+1]LTC[CAM]TLTGLR.+4b12+2.light	32
741.901028	966.015272	15	sp|P01876|IGHA1_HUMAN.LSLHRPALEDLLLGSEAN[+1]LTC[CAM]TLTGLR.+4b18+2.light	32
744.403095	830.442818	15	sp|P01876|IGHA1_HUMAN.LSLHRPALEDLLLGSEAN[+1]LTC[CAM]TLTGLR.+4y7.heavy	32
744.403095	888.541397	15	sp|P01876|IGHA1_HUMAN.LSLHRPALEDLLLGSEAN[+1]LTC[CAM]TLTGLR.+4b8.heavy	32
744.403095	679.893169	15	sp|P01876|IGHA1_HUMAN.LSLHRPALEDLLLGSEAN[+1]LTC[CAM]TLTGLR.+4b12+2.heavy	32
744.403095	966.015272	15	sp|P01876|IGHA1_HUMAN.LSLHRPALEDLLLGSEAN[+1]LTC[CAM]TLTGLR.+4b18+2.heavy	32
600.004686	658.413403	15	sp|P00738|HPT_HUMAN.VVLHPN[DeW]YSQVDIGLIK.+3y6.light	22
600.004686	800.434978	15	sp|P00738|HPT_HUMAN.VVLHPN[DeW]YSQVDIGLIK.+3y14+2.light	22
600.004686	1041.524967	15	sp|P00738|HPT_HUMAN.VVLHPN[DeW]YSQVDIGLIK.+3b9.light	22
600.004686	570.800328	15	sp|P00738|HPT_HUMAN.VVLHPN[DeW]YSQVDIGLIK.+3b10+2.light	22
599.008599	658.413403	15	sp|P00738|HPT_HUMAN.VVLHPNYSQVDIGLIK.+3y6.light	22
599.008599	798.940847	15	sp|P00738|HPT_HUMAN.VVLHPNYSQVDIGLIK.+3y14+2.light	22
599.008599	1038.536706	15	sp|P00738|HPT_HUMAN.VVLHPNYSQVDIGLIK.+3b9.light	22
599.008599	569.306198	15	sp|P00738|HPT_HUMAN.VVLHPNYSQVDIGLIK.+3b10+2.light	22
601.679999	666.427602	15	sp|P00738|HPT_HUMAN.VVLHPNYSQVDIGLIK.+3y6.heavy	22
601.679999	802.947947	15	sp|P00738|HPT_HUMAN.VVLHPNYSQVDIGLIK.+3y14+2.heavy	22
601.679999	1038.536706	15	sp|P00738|HPT_HUMAN.VVLHPNYSQVDIGLIK.+3b9.heavy	22
601.679999	569.306198	15	sp|P00738|HPT_HUMAN.VVLHPNYSQVDIGLIK.+3b10+2.heavy	22
599.336605	658.413403	15	sp|P00738|HPT_HUMAN.VVLHPN[+1]YSQVDIGLIK.+3y6.light	22
599.336605	799.432855	15	sp|P00738|HPT_HUMAN.VVLHPN[+1]YSQVDIGLIK.+3y14+2.light	22
599.336605	1039.520722	15	sp|P00738|HPT_HUMAN.VVLHPN[+1]YSQVDIGLIK.+3b9.light	22
599.336605	569.798206	15	sp|P00738|HPT_HUMAN.VVLHPN[+1]YSQVDIGLIK.+3b10+2.light	22
602.008004	666.427602	15	sp|P00738|HPT_HUMAN.VVLHPN[+1]YSQVDIGLIK.+3y6.heavy	22
602.008004	803.439955	15	sp|P00738|HPT_HUMAN.VVLHPN[+1]YSQVDIGLIK.+3y14+2.heavy	22
602.008004	1039.520722	15	sp|P00738|HPT_HUMAN.VVLHPN[+1]YSQVDIGLIK.+3b9.heavy	22

602.008004	569.798206	15	sp|P00738|HPT_HUMAN.VVLHPN[+1]YSQVDIGLIK.+3b10+2.heavy	22
1125.879312	915.424044	15	sp|P04003|C4BPA_HUMAN.FSLLGHASISC[CAM]TVEN[DeW]ETIGVWRPSPPTC[CAM]EK.+3y8.light	55
1125.879312	1182.04824	15	sp|P04003|C4BPA_HUMAN.FSLLGHASISC[CAM]TVEN[DeW]ETIGVWRPSPPTC[CAM]EK.+3y20+2.light	55
1125.879312	1010.482527	15	sp|P04003|C4BPA_HUMAN.FSLLGHASISC[CAM]TVEN[DeW]ETIGVWRPSPPTC[CAM]EK.+3b19+2.light	55
1125.879312	1231.106946	15	sp|P04003|C4BPA_HUMAN.FSLLGHASISC[CAM]TVEN[DeW]ETIGVWRPSPPTC[CAM]EK.+3b22+2.light	55
844.661303	915.424044	15	sp|P04003|C4BPA_HUMAN.FSLLGHASISC[CAM]TVEN[DeW]ETIGVWRPSPPTC[CAM]EK.+4y8.light	40
844.661303	1001.974869	15	sp|P04003|C4BPA_HUMAN.FSLLGHASISC[CAM]TVEN[DeW]ETIGVWRPSPPTC[CAM]EK.+4y17+2.light	40
844.661303	937.453573	15	sp|P04003|C4BPA_HUMAN.FSLLGHASISC[CAM]TVEN[DeW]ETIGVWRPSPPTC[CAM]EK.+4y16+2.light	40
844.661303	707.350811	15	sp|P04003|C4BPA_HUMAN.FSLLGHASISC[CAM]TVEN[DeW]ETIGVWRPSPPTC[CAM]EK.+4y12+2.light	40
1124.883225	915.424044	15	sp|P04003|C4BPA_HUMAN.FSLLGHASISC[CAM]TVENETIGVWRPSPPTC[CAM]EK.+3y8.light	55
1124.883225	1180.554109	15	sp|P04003|C4BPA_HUMAN.FSLLGHASISC[CAM]TVENETIGVWRPSPPTC[CAM]EK.+3y20+2.light	55
1124.883225	1008.988397	15	sp|P04003|C4BPA_HUMAN.FSLLGHASISC[CAM]TVENETIGVWRPSPPTC[CAM]EK.+3b19+2.light	55
1124.883225	1229.612816	15	sp|P04003|C4BPA_HUMAN.FSLLGHASISC[CAM]TVENETIGVWRPSPPTC[CAM]EK.+3b22+2.light	55
1127.554625	923.438243	15	sp|P04003|C4BPA_HUMAN.FSLLGHASISC[CAM]TVENETIGVWRPSPPTC[CAM]EK.+3y8.heavy	55
1127.554625	1184.561209	15	sp|P04003|C4BPA_HUMAN.FSLLGHASISC[CAM]TVENETIGVWRPSPPTC[CAM]EK.+3y20+2.heavy	55
1127.554625	1008.988397	15	sp|P04003|C4BPA_HUMAN.FSLLGHASISC[CAM]TVENETIGVWRPSPPTC[CAM]EK.+3b19+2.heavy	55
1127.554625	1229.612816	15	sp|P04003|C4BPA_HUMAN.FSLLGHASISC[CAM]TVENETIGVWRPSPPTC[CAM]EK.+3b22+2.heavy	55
843.914238	915.424044	15	sp|P04003|C4BPA_HUMAN.FSLLGHASISC[CAM]TVENETIGVWRPSPPTC[CAM]EK.+4y8.light	40
843.914238	1000.480739	15	sp|P04003|C4BPA_HUMAN.FSLLGHASISC[CAM]TVENETIGVWRPSPPTC[CAM]EK.+4y17+2.light	40
843.914238	935.959442	15	sp|P04003|C4BPA_HUMAN.FSLLGHASISC[CAM]TVENETIGVWRPSPPTC[CAM]EK.+4y16+2.light	40
843.914238	707.350811	15	sp|P04003|C4BPA_HUMAN.FSLLGHASISC[CAM]TVENETIGVWRPSPPTC[CAM]EK.+4y12+2.light	40
845.917788	923.438243	15	sp|P04003|C4BPA_HUMAN.FSLLGHASISC[CAM]TVENETIGVWRPSPPTC[CAM]EK.+4y8.heavy	40
845.917788	1004.487838	15	sp|P04003|C4BPA_HUMAN.FSLLGHASISC[CAM]TVENETIGVWRPSPPTC[CAM]EK.+4y17+2.heavy	40
845.917788	939.966542	15	sp|P04003|C4BPA_HUMAN.FSLLGHASISC[CAM]TVENETIGVWRPSPPTC[CAM]EK.+4y16+2.heavy	40
845.917788	711.35791	15	sp|P04003|C4BPA_HUMAN.FSLLGHASISC[CAM]TVENETIGVWRPSPPTC[CAM]EK.+4y12+2.heavy	40
1125.211231	915.424044	15	sp|P04003|C4BPA_HUMAN.FSLLGHASISC[CAM]TVEN[+1]ETIGVWRPSPPTC[CAM]EK.+3y8.light	55
1125.211231	1181.046117	15	sp|P04003|C4BPA_HUMAN.FSLLGHASISC[CAM]TVEN	55

			[+1]ETIGVWRPSPPTC[CAM]EK.+3y20+2.light	
1125.211231	1009.480405	15	sp|P04003|C4BPA_HUMAN.FSLLGHASISC[CAM]TVEN [+1]ETIGVWRPSPPTC[CAM]EK.+3b19+2.light	55
1125.211231	1230.104824	15	sp|P04003|C4BPA_HUMAN.FSLLGHASISC[CAM]TVEN [+1]ETIGVWRPSPPTC[CAM]EK.+3b22+2.light	55
1127.88263	923.438243	15	sp|P04003|C4BPA_HUMAN.FSLLGHASISC[CAM]TVEN [+1]ETIGVWRPSPPTC[CAM]EK.+3y8.heavy	55
1127.88263	1185.053217	15	sp|P04003|C4BPA_HUMAN.FSLLGHASISC[CAM]TVEN [+1]ETIGVWRPSPPTC[CAM]EK.+3y20+2.heavy	55
1127.88263	1009.480405	15	sp|P04003|C4BPA_HUMAN.FSLLGHASISC[CAM]TVEN [+1]ETIGVWRPSPPTC[CAM]EK.+3b19+2.heavy	55
1127.88263	1230.104824	15	sp|P04003|C4BPA_HUMAN.FSLLGHASISC[CAM]TVEN [+1]ETIGVWRPSPPTC[CAM]EK.+3b22+2.heavy	55
844.160242	915.424044	15	sp|P04003|C4BPA_HUMAN.FSLLGHASISC[CAM]TVEN [+1]ETIGVWRPSPPTC[CAM]EK.+4y8.light	40
844.160242	1000.972747	15	sp|P04003|C4BPA_HUMAN.FSLLGHASISC[CAM]TVEN [+1]ETIGVWRPSPPTC[CAM]EK.+4y17+2.light	40
844.160242	936.45145	15	sp|P04003|C4BPA_HUMAN.FSLLGHASISC[CAM]TVEN [+1]ETIGVWRPSPPTC[CAM]EK.+4y16+2.light	40
844.160242	707.350811	15	sp|P04003|C4BPA_HUMAN.FSLLGHASISC[CAM]TVEN [+1]ETIGVWRPSPPTC[CAM]EK.+4y12+2.light	40
846.163792	923.438243	15	sp|P04003|C4BPA_HUMAN.FSLLGHASISC[CAM]TVEN [+1]ETIGVWRPSPPTC[CAM]EK.+4y8.heavy	40
846.163792	1004.979846	15	sp|P04003|C4BPA_HUMAN.FSLLGHASISC[CAM]TVEN [+1]ETIGVWRPSPPTC[CAM]EK.+4y17+2.heavy	40
846.163792	940.45855	15	sp|P04003|C4BPA_HUMAN.FSLLGHASISC[CAM]TVEN [+1]ETIGVWRPSPPTC[CAM]EK.+4y16+2.heavy	40
846.163792	711.35791	15	sp|P04003|C4BPA_HUMAN.FSLLGHASISC[CAM]TVEN [+1]ETIGVWRPSPPTC[CAM]EK.+4y12+2.heavy	40

The transitions for the C4BPA_HUMAN protein were excluded in this study, since the deaminated and non-deaminated peptides could not be resolved chromatographically, which is used as a critical criterion for integrating the corresponding MRM-transition signals. The entries however remained in the method file, contributing to the total cycle time during sample acquisition. For reasons of completeness and integrity of the method description, these transitions remain listed in the MRM transition table.

Supplemental Table 2 ^{16}O deamination reactions

Sample	ID Nr	TRFE_HUMAN average	SEM	IGHA1_HUMAN average	SEM	HPT_HUMAN average	SEM
PMM2-CDG	001	5.53	0.64	18.30	2.80	0.70	0.70
PMM2-CDG	002	7.40	1.59	16.57	1.02	2.20	1.14
PMM2-CDG	003	5.63	0.63	13.53	0.98	2.50	1.29
PMM2-CDG	004	5.47	0.96	12.70	0.23	1.73	0.18
PMM2-CDG	005	4.97	0.28	13.53	2.09	1.17	0.15
AlG11-CDG	034	4.93	0.07	9.40	2.86	2.47	1.39
AlLG1-CDG	036	6.70	0.87	9.10	0.06	2.67	2.67
ALG6-CDG	037	4.00	0.32	6.60	0.10	0.00	0.00
MPDU1-CDG	038	5.67	0.32	6.20	0.23	0.00	0.00
RFT1-CDG	039	5.43	0.26	8.73	1.84	1.53	0.78
healthy	007	7.37	0.93	16.00	1.76	0.00	0.00
healthy	008	5.67	0.50	13.87	1.62	5.93	0.96
healthy	009	6.53	1.11	14.70	1.30	6.37	2.75
healthy	010	5.40	0.45	15.97	1.34	2.50	2.50
healthy	011	6.03	1.63	14.50	4.26	3.17	3.17
Steatosis	012	5.23	1.45	14.50	1.88	4.87	0.38
Steatosis	013	5.63	0.50	14.53	0.96	6.50	0.46
Steatosis	015	4.73	0.69	15.90	3.95	4.97	0.09
Steatosis	016	4.67	0.48	12.57	1.38	1.47	1.47
NASH	014	4.77	0.23	14.30	1.50	2.03	2.03
NASH	017	4.93	0.75	13.57	0.90	2.90	1.46
NASH	018	4.43	1.03	14.00	1.95	2.53	1.75
NASH	019	4.80	0.65	17.83	1.35	4.83	0.86
NASH	020	4.40	1.28	12.00	2.61	1.57	1.57
NASH	021	5.33	0.81	12.07	3.34	3.50	1.92
NASH	022	4.43	1.03	9.00	1.54	4.23	2.14
NASH	023	5.73	0.42	9.33	2.31	5.70	3.69
**Total average**		**5.40**	**0.74**	**12.94**	**1.71**	**2.89**	**1.31**

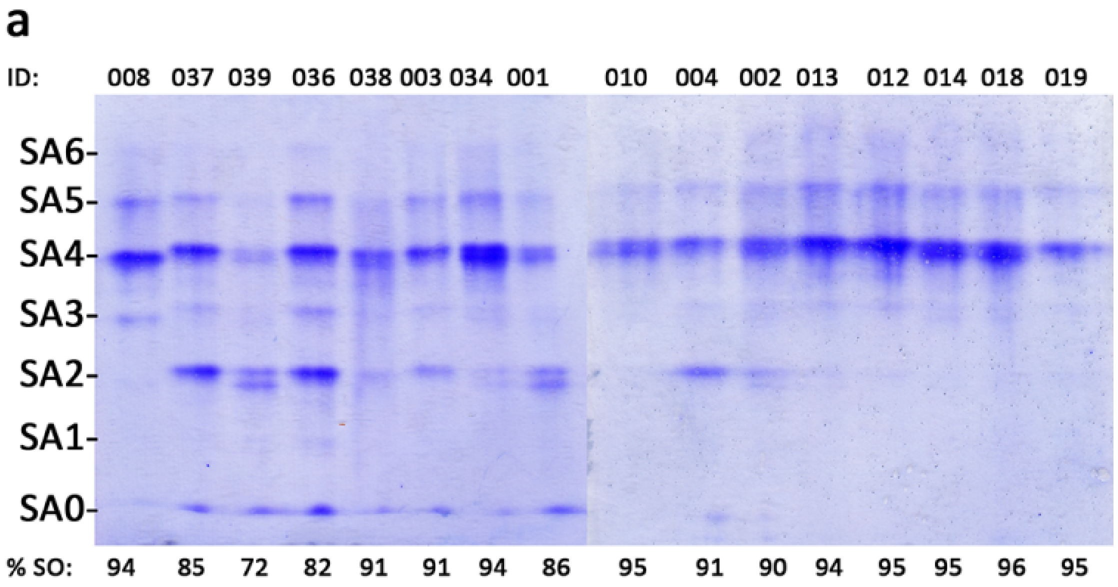

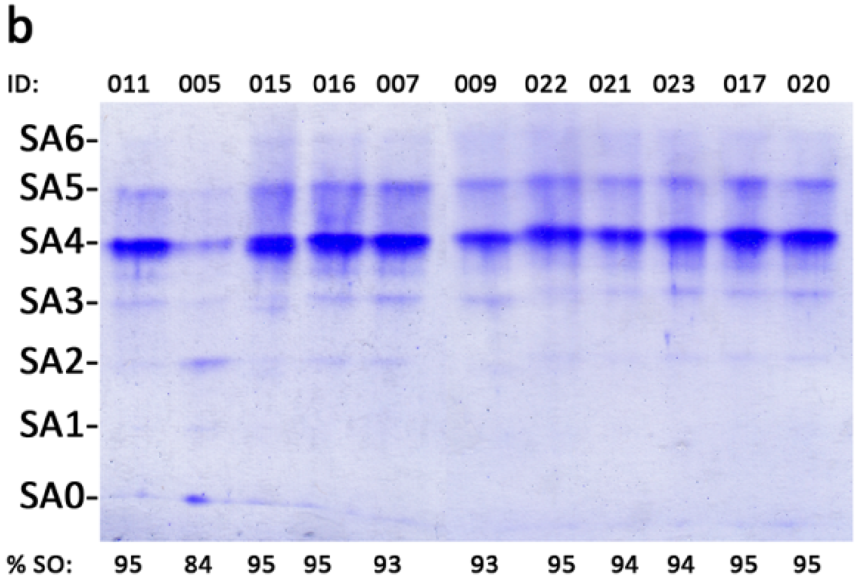

Supplemental Figure 1. Isoelectric focusing gel electrophoresis of transferrin of the samples analyzed.

Panel **a**: ID 008 and 010, healthy control serum; ID 037, ALG6-CDG; ID 039, RFT1-CDG; ID 036, Alg1-CDG; ID 038, MPDU1-CDG; ID 003, PMM2-CDG; ID 034, ALG11-CDG; ID 001, PMM2-CDG; ID 004 and 002, PMM2-CDG; ID 013 and 012, steatosis; ID014-019, NASH. Panel **b**: ID 011, 007 and 009 healthy control serum ; ID 005, PMM2-CDG; ID 015 and 016, steatosis; ID 022-020, NASH. SA0-SA6 indicate the number of sialic acids on the transferrin N-glycans. The percent site occupancy of the corresponding transferrin glycopeptide is indicated below the lanes (% SO = percent site occupancy, ID = unique sample identifier).

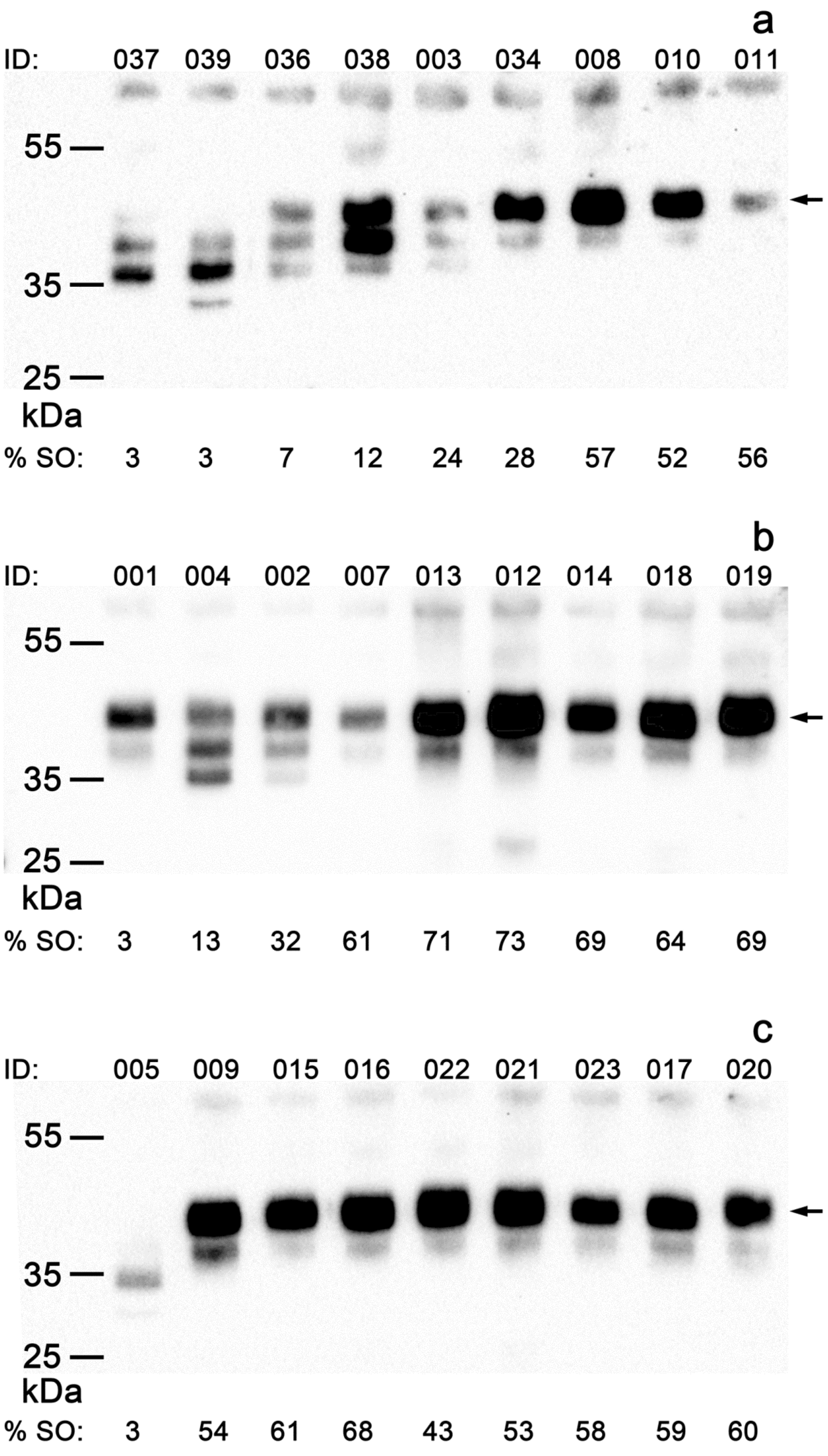
a
ID: 037 039 036 038 003 034 008 010 011
55
35
25
kDa
% SO: 3 3 7 12 24 28 57 52 56
b
ID: 001 004 002 007 013 012 014 018 019
55
35
25
kDa
% SO: 3 13 32 61 71 73 69 64 69
c
ID: 005 009 015 016 022 021 023 017 020
55
35
25
kDa
% SO: 3 54 61 68 43 53 58 59 60

Supplemental Figure 2. Western-blot analyses of haptoglobin of the samples analyzed.

Panel **a**: ID 037, ALG6-CDG; ID 039, RFT1-CDG; ID 036, Alg1-CDG; ID 038, MPDU1-CDG; ID 003, PMM2-CDG; ID 034, ALG11-CDG; ID 008-011, healthy control serum. Panel **b**: ID 001-002, PMM2-CDG; ID 007, healthy control serum; ID 013-012, steatosis; ID 014-019, NASH. Panel **c**: ID 005, PMM2-CDG, ID 009, healthy control serum; ID 015-016, steatosis; ID 022-020, NASH. The arrows indicate the migration positions of fully glycosylated haptoglobin. The migration positions of the protein molecular weight markers are indicated at the left in kDa. The percent site occupancy of the corresponding haptoglobin glycopeptide is indicated below the lanes (% SO = percent site occupancy, ID = unique sample identifier)

The haptoglobin Western blot banding of ID 001 does not agree with a PMM2-CDG phenotype. The same sample however shows a typical isoelectric focusing pattern as observed with PMM2-CDG with increased proportions of SA2 and SA0 bands (Supplemental Figure 1). Furthermore, the site occupancy for the haptoglobin peptide referring to sample ID 001 was determined to be 3 % by MRM-MS (Table 2), indicating a severe clinical presentation of the PMM2-CDG patient. The isoelectric focusing gel electrophoresis was performed as described earlier.[1]

1 Hülsmeier, A. J., Paesold-Burda, P. & Hennet, T. N-glycosylation site occupancy in serum glycoproteins using multiple reaction monitoring liquid chromatography-mass spectrometry. *Mol Cell Proteomics* **6**, 2132-2138, doi:10.1074/mcp.M700361-MCP200 (2007).

Supplemental Table 3 Statistical comparison of the CDG and NAFLD disease groups

Disease groups	Two-tailed P values			
	TRFE_HUMAN	HPT_HUMAN	IGHA1_HUMAN	IGHG2_HUMAN
CDG severe versus CDG moderate	0.7143	0.0571	0.8571	> 0.9999
CDG moderate versus CDG mild	0.7000	0.1000	0.4000	> 0.9999
Steatosis versus NASH	0.8485	0.0727	0.1455	> 0.9999

Differences between groups were calculated using the Mann-Whitney statistical test, and were considered significant with $p < 0.05$.

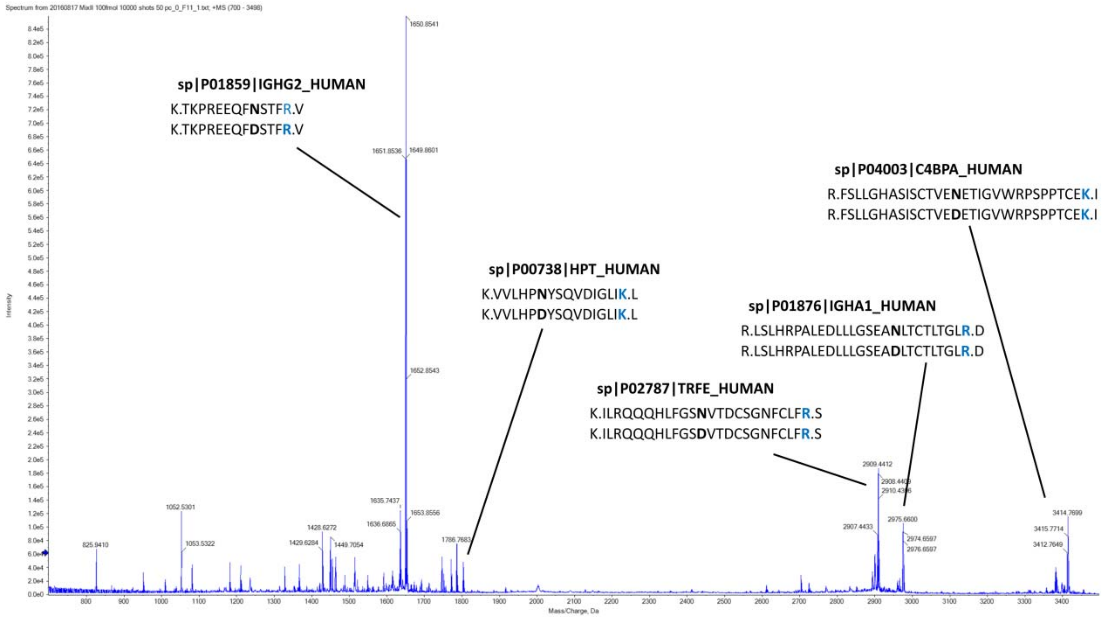

Supplemental Figure 3. MALDI-TOF-MS of the standard peptide mix consisting of the synthetic peptides with c-terminal labeled arginine or lysine (bold blue letters) corresponding to the target glycopeptide sequences. The glycosylation site amino acid is marked bold.
